# Indocyanine Green-Loaded Halloysite Nanotubes as Photothermal Agents

**DOI:** 10.1021/acsomega.3c03268

**Published:** 2023-10-03

**Authors:** Oyku Demirel, Selin Oyku Gundogdu, Sena Yuce, Hayriye Unal

**Affiliations:** † Faculty of Engineering and Natural Sciences, Sabanci University, Istanbul 34956, Turkey; ‡ SUNUM Nanotechnology Research Center, Sabanci University, Istanbul 34956, Turkey

## Abstract

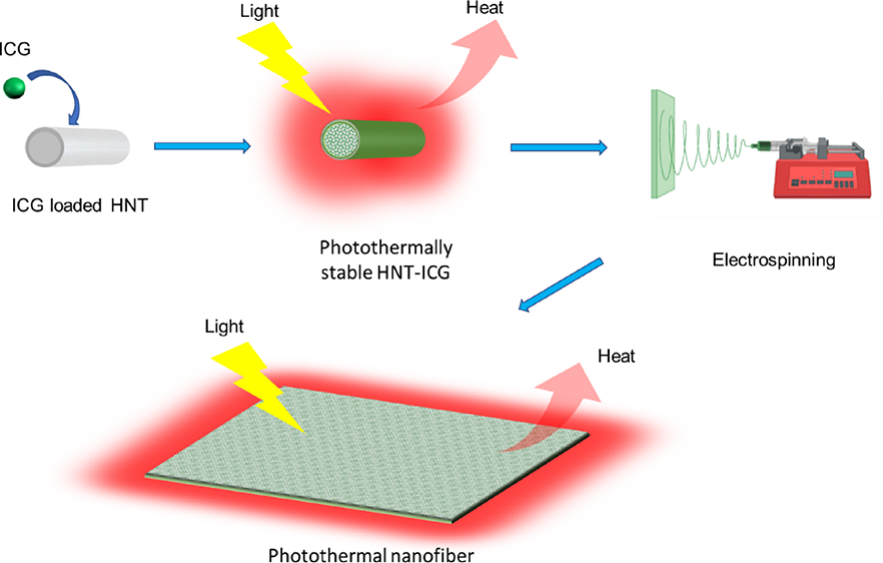

Photothermal nanoparticles with light-to-heat conversion properties have gained interest in recent years and have been used in a variety of applications. Herein, indocyanine green (ICG), which is commonly employed as a photothermal agent suffering from low photostability, was loaded into halloysite nanotubes (HNTs) resulting in photothermal HNT-ICG nanohybrids. The photothermal heating patterns of the prepared photothermal nanohybrids as a result of near-infrared (NIR) irradiation were carefully examined. The nanohybrids reached a temperature of 216 °C in 2 min under NIR light, and in contrast to free NIR, the ICG loaded into HNTs remained stable over 10 heating and cooling cycles. Moreover, HNT-ICG nanohybrids incorporated into polyacrylonitrile (PAN) were electrospun into nanofibers for use as photothermal nanofibers, and composite nanofibers, which heat up to 79.3 °C under 2 min of NIR irradiation, were obtained. To demonstrate the potential of the PAN/HNT-ICG nanofibers as light-activated antibacterial nanofibers, their NIR light-activated killing activity on **Staphylococcus aureus** (**S. aureus**) cells has been explored. The composite nanofibers reduced the number of bacteria on their surface by 7log upon 10 min of NIR irradiation. Encapsulation of ICG in HNTs as a carrier has been demonstrated as an effective way to stabilize ICG and incorporate it into materials and coatings without compromising its functionality.

## Introduction

Photothermal nanoparticles, which generate local heat when exposed to NIR light, have attracted tremendous attention.^1^ In recent years, light-activated heat generation using photothermal nanoparticles has had a wide range of unique applications such as cancer therapy, drug delivery, water evaporation, and antimicrobial therapy.^2−6^ Numerous photothermal agents such as metallic nanoparticles including metal oxide, gold, copper, platinum, and palladium;^7−9^ carbon-based nanoparticles including carbon nanotubes and reduced graphene oxide;^10−12^ polymers including polydopamine and polyaniline;^13,14^ and organic dyes including cyanine dyes^15−17^ have been extensively studied. Among them, organic dyes, which are safe, biocompatible, and tunable and have strong NIR absorbance, came to the fore and have been widely investigated.^18^

One of the typical representatives of NIR dyes is ICG.^19^ ICG is a water-soluble, nontoxic, photoactivated fluorescent iodide—a synthetic organic dye that is excited by NIR light between 780 and 820 nm and belongs to the group of NIR-active cyanine dyes.^20,21^ It first appeared in applications in the mid-1950s and is still a hot topic in numerous areas recently.^19^ Due to its unique properties such as potential biodegradability in biological systems, strong NIR absorption/fluorescent emission, and nontoxicity, it has been utilized as a fluorescence contrast agent for imaging purposes such as optical spectroscopy and tomography.^22^ Furthermore, when exposed to NIR light, the ICG converts the majority of the excitation energy into heat, causing local heating that allows its employment for photothermal therapy.^23^ However, the photodegradation, thermal degradation, limited reusability, and aqueous-instability properties of ICG adversely affect its photothermal efficiency in these applications.^24−26^ One of the most significant approaches to overcome the abovementioned disadvantages of ICG has been its impregnation in nanosized carriers.^24,27^ By incorporating ICG into nanoparticles, hybrid nanomaterials, which address the limitations of ICG, were created.^28^ For instance, ICG self-assembled with phospholipid-polyethylene glycol (PL–PEG) showed higher stability than free ICG after NIR irradiation for tumor suppression via photothermal therapy.^29^ ICG was integrated into photothermal network-based thermosensitive hydrogel, a supramolecular cross-linked conjugated polymer, for photothermal therapy applications, and the NIR-modulated hydrogel platform has been shown to increase the stability of ICG.^30^ Another approach toward stabilization of the ICG has been its incorporation into electrospun nanofibers as nanocarriers. ICG incorporated into poly(vinyl alcohol) nanofibers was shown to be a promising new approach for the utilization of ICG in photodynamic antimicrobial chemotherapy.^31^

In this study, a new photothermal nanoparticle with strong light-to-heat conversion properties was developed by impregnating HNTs with ICG. HNTs, which have attractive properties such as biocompatibility, low cost, hollow tubular structure, and ease of modification, and have been successfully utilized in the literature for different applications ranging from mechanical reinforcement of composites to selective nano functionalization.^32−34^ Herein, HNTs were used as nanocarriers to encapsulate ICG, resulting in photothermal nanohybrids. The stability of the photothermal properties of ICG impregnated in HNTs and the light-activated heating properties of the HNT-ICG nanohybrids were studied. As a demonstration of potential applications of the HNT-ICG nanohybrids as photothermal agents, electrospun nanofibers containing HNT-ICG nanohybrids and their photothermal properties were presented.

## Experimental Section

### Chemicals

HNTs were supplied by Esan Eczacıbaşı (Turkey). ICG and ultrapure Tris Base (Tris-hydroxymethyl aminomethane) were purchased from MP Biomedicals, LLC. Polyacrylonitrile (PAN) (MW = 150 kDa) and *N*,*N*-dimethylformamide (DMF) (99.8%) were purchased from Sigma-Aldrich. Tryptic soy broth (TSB) and agar powder were purchased from Medimark (Italy). Antibacterial activity tests were carried out using **Staphylococcus aureus** (**S. aureus**) (ATCC 29213). All experiments were performed by using deionized (DI) water.

### Preparation of HNT-ICG Nanohybrids

ICG loading into HNTs was carried out by vacuum-assisted loading.^35^ HNTs were added to DI water at a concentration of 10 mg/mL, and the mixture was ultrasonicated with a probe sonicator (QSonica, Q700, Newtown, Connecticut) for 10 min in an ice bath with a 5 s pulse on and a 2 s pulse off to obtain an aqueous dispersion. The ICG was dissolved in DI water at a concentration of 1 mg/mL and mixed with the HNT dispersion in various weight ratios (0.2, 0.5, 2, and 10 wt %). Consequently, the final ICG concentrations in the aqueous HNT/ICG dispersion before the loading were 1, 0.2, 0.05, and 0.02 mg/mL for the preparation of HNT-ICG_10 HNT-ICG_2, HNT-ICG_0.5, and HNT-ICG_0.2 nanohybrids, respectively. To load the ICG molecules into the inner cavity of the HNT, the dispersion was vacuumed at a 1 mbar pressure for 20 min, and the air in the HNTs was evacuated. The vacuum procedure was repeated twice to load the ICG molecules into the HNTs. Afterward, the ICG-loaded HNTs (HNT-ICG) were washed and centrifuged at 10,000 rpm to remove excess ICG molecules. The washing was repeated five times. The produced nanohybrids were dried overnight at 50 °C.

### Characterization of HNT-ICG Nanohybrids

The TECAN Infinite F200 microplate reader spectrophotometer was used to determine the actual amount of ICG molecules loaded into the HNTs. The supernatant and rinse samples of the HNT-ICG nanohybrids obtained after the vacuum application were placed in a 96-well plate, and absorbance spectra between 550 and 900 nm were recorded with stirring at 21 °C. To determine the concentration of the ICG in the supernatant solution, a standard curve was constructed with aqueous ICG solutions at different concentrations between 0.05 and 100 μM. The % ICG loading in HNTs and loading efficiency were calculated using eqs 1 and 2, respectively.
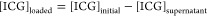
1
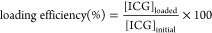
2where [ICG]_loaded_ is the concentration of ICG integrated into the HNTs and [ICG]_initial_ is the initial concentration of ICG used in the loading process, and [ICG]_supernatant_ is the ICG concentration of the supernatant solution obtained after the loading calculated from the standard curve, which is the concentration of free ICG molecules that could not be integrated into the HNTs during the process.

Thermogravimetric analysis (TGA) (Shimadzu Corp. DTG-60H (TGA/DTA)) was performed to determine the ICG content of the nanohybrids. TGA was carried out under nitrogen flow at a scanning range of 30 to 1000 °C and a heating rate of 10 °C/min.

To study the release of ICG from HNT-ICG nanohybrids, HNT-ICG nanohybrids were dispersed in water at a concentration of 1 mg/mL. The dispersion was incubated in the dark by stirring with a magnetic stirrer at 400 rpm for 24 h at room temperature. Samples were taken from the dispersion at different time intervals and centrifuged to remove the HNT-ICG nanohybrids, and the absorbance of the supernatant was recorded with the TECAN plate reader.

### Photothermal Properties of the HNT-ICG Nanohybrids

Photothermal properties of the HNT-ICG nanohybrids were studied by constructing time–temperature profiles of the HNT-ICG nanohybrids in powder form under NIR irradiation. HNT-ICG powder (0.5 g) was placed in a Teflon holder and irradiated with an 808 nm laser module (STEMINC, SMM22808E1200) (Doral, Florida, USA) at 0.8 W/cm^2^; the temperature of the HNT-ICG powder was monitored with a FLIR E6xt thermal camera. The HNT-ICG_10 sample was exposed to NIR laser light for 2 min, followed by the switching off the light source for 2 min for cooling at room temperature. The irradiation on/off cycle was repeated 10 times consecutively for the photothermal analysis.

### Photothermal Stability of the HNT-ICG Nanohybrids

The photostability of the aqueous ICG solution and HNT-ICG nanohybrids was tested. ICG was dissolved in water at a concentration of 0.025 mg/mL. HNT-ICG_10 nanohybrids (0.25 g) were dispersed in water by ultrasonication for 20 min with 5-pulse on and 2-pulse off. The aqueous ICG solution and the aqueous HNT-ICG dispersions were irradiated with an 808 nm laser module (STEMINC, SMM22808E1200) (Doral, Florida, USA) for 10 min at 0.8 W/cm^2^ light density, followed by switching of the laser light for 5 min to allow the samples to cool. Three irradiation/cooling cycles, during which the temperature was monitored with an FLIR E6xt thermal camera, were performed. Absorbance spectra of the nonirradiated samples along with the samples irradiated three times were recorded between 550 and 900 nm and were recorded on a TECAN plate reader with stirring at 21 °C.

### Preparation of PAN/HNT-ICG Nanofibers

The final PAN/HNT-ICG electrospinning solution consisted of PAN (7 wt % in DMF) and 50 wt % HNT-ICG_10 nanohybrid. PAN in DMF was stirred with a magnetic bar at 500 rpm for 2 days at room temperature. Then, the HNT-ICG_10 nanohybrid was added into the prepared dispersion and stirred under the same conditions for 10 min to obtain a homogeneous distribution. The PAN/ICG electrospinning solution for the control nanofiber contained 5 wt % free ICG in aqueous solution.

PAN/HNT-ICG and PAN/ICG nanofibers were produced by using the electrospinning system (Inovenso starter kit) with a syringe pump, a vertical metal plate collector, and a DC voltage power supply. PAN/HNT-ICG and PAN/ICG electrospinning solutions were loaded into a 5 mL syringe with a 13.10 mm diameter and a stainless-steel needle. A 15 × 15 cm electrically grounded metal plate wrapped in aluminum foil was placed 20 cm ahead of the needle tip. The applied voltage and flow rate of the solution were set to 12 kV and 1 mL/h, respectively. Electrospinning was carried out for a total of 5 h.

### Characterization of PAN/HNT-ICG Nanofibers

HNT-ICG content of the nanofiber was determined by TGA (Shimadzu Corp. DTG-60H (TGA/DTA)). ICG powder and PAN, PAN/ICG, and PAN/HNT-ICG nanofibers were analyzed under a nitrogen flow, with a scanning range of 30 to 1000 °C and a heating rate of 10 °C/min.

Experimental % weight of ICG in the PAN/ICG nanofibers was calculated by determining the weight change difference of PAN and PAN/ICG at 1000 °C. Experimental % weight of ICG in the PAN/HNT-ICG nanofibers was calculated by determining the weight change difference of PAN/HNT-ICG and HNT-ICG at 1000 °C and normalizing this difference by the remaining weight of HNT at this temperature.

The surface morphology and diameter of the PAN/HNT-ICG nanofibers were examined using a Zeiss Leo Supra 35VP scanning electron microscope (SEM). Samples were coated with Au–Pd, and images were collected at 2 kV by using the secondary electron detector.

### Photothermal Properties of PAN/HNT-ICG Nanofibers

To investigate the photothermal properties of the nanofibers, we constructed their time–temperature profiles under NIR irradiation. PAN/HNT-ICG nanofibers (1 × 1 cm) were placed in a Teflon holder and irradiated with an 808 nm laser module at 0.8 W/cm^2^ (STEMINC, SMM22808E1200) (Doral, Florida, USA) for 2 min. The light source was switched off after 2 min to cool the samples to room temperature. Irradiation/cooling cycles were repeated 10 times, during which temperatures were recorded by using an FLIR E6xt thermal camera.

### Light-Activated Antibacterial Properties of Nanofibers

*S. aureus* (ATCC 29213) cells were cultured for 24 h in 3 mL TSB growth medium at 37 °C in an incubator with 200 rpm shaking. Grown bacteria were centrifuged, washed twice with sterile Tris buffer (pH 7.5), and resuspended at a concentration of 10^8^ CFU/mL in Tris buffer. Nanofibers cut to 1 × 1 cm were placed in the wells of Teflon molds sized 1 cm in diameter and 0.5 cm in height. 100 μL of bacterial suspension was added to the well containing the nanofiber; the nanofiber surface was totally covered. To ensure that bacteria were not disrupted by NIR light alone, the same amount of bacterial suspension was introduced to an empty well as a control. For each fiber type, two samples were prepared. To examine the antibacterial properties of nanofibers activated by NIR light, one was exposed to NIR light with an 808 nm laser module at 0.8 W/cm^2^ (STEMINC, SMM22808E1200) (Doral, Florida, USA) for varied durations of time (2 and 4 min) while the other was left in the dark for the same duration as a control. Each of the light-treated and control nanofibers received 1 mL of Tris buffer. To transfer the bacteria to the solution, the suspension was vortexed for 2 min. Serially diluted bacterial suspensions were plated on TSB agar plates and incubated at 37 °C for 24 h, and colonies were counted. The viability of **S. aureus** was reported as log_10_ CFU/mL. Three independent experiments’ mean and standard error values are presented.

## Results and Discussion

**Figure 1:**
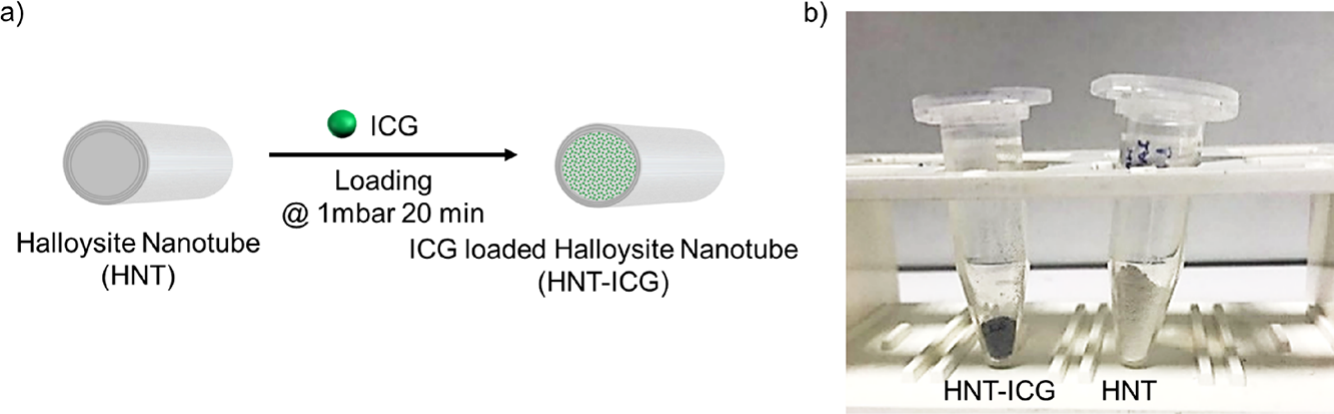
(a) Loading of HNTs with ICG. (b) HNT-ICG (left) and HNT (right) powder.

ICG molecules were loaded into the lumen of the HNT clay nanoparticles to obtain HNT-ICG nanohybrids (Figure 1a). Vacuum application to the mixture of HNTs in an aqueous ICG solution evacuates the lumen of HNTs and allows the loading of the ICG molecules upon termination of the vacuum. Centrifugation of the mixture and subsequent drying yielded HNT-ICG nanohybrids in the form of a dark blue-greenish powder (Figure 1b). The color alteration indicated the successful loading of ICG molecules into HNTs.

**Figure 2:**
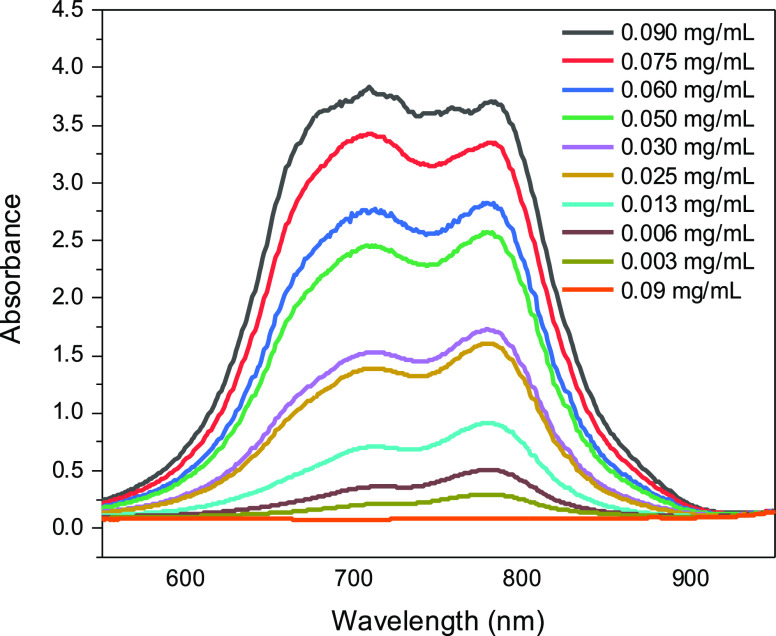
Absorbance spectra of aqueous ICG solutions at 0 to 0.09 mg/mL concentrations.

HNT-ICG nanohybrids were prepared at different ICG concentrations to analyze whether the light-activated heating properties of the resulting HNT-ICG nanohybrids could be controlled by the amount of ICG loaded into HNTs. The experimental ratio by weight of loaded ICG molecules was calculated by determining the ICG content of the supernatant solution obtained following the vacuum application, namely, by determining the amount of ICG not loaded into the HNTs utilizing the specific absorbance of ICG at 780 nm.^17^ The concentration of the supernatant solution was calculated via a standard curve constructed from the absorbance values of aqueous ICG solutions prepared at 0 to 0.09 mg/mL (Figure 2). The absorbance values of the ICG solutions at increasing concentrations clearly demonstrate that H-dimers of ICG molecules are being formed as the solution concentration increased, as indicated by the absorbance max centered at 700 nm, which is specific for ICG H-dimers.^36^ By looking at the absorbance spectra obtained at different concentrations, it can be concluded that the ICG molecules were mostly in H-dimer forms before being loaded into HNTs. The weight ratio of ICG molecules in the HNT-ICG nanohybrids along with the loading efficiency is presented in Table 1. While all ICG molecules were loaded into HNTs at 0.2 and 0.5 wt % ICG concentrations, the loading efficiencies were 92.4 and 87.9%, at 2 and 10 wt % ICG concentrations, respectively. This demonstrated that the loading capacity of the HNTs was reached at higher ICG concentrations and the maximum loading was obtained in HNT-ICG_10 nanohybrids.

**Table 1:** ICG Content and Loading Efficiency of the HNT-ICG Nanohybrids

sample name	ICG loading (wt %)	efficiency (%)
HNT-ICG_0.2	0.2	100.0
HNT-ICG_0.5	0.5	100.0
HNT-ICG_0.2	1.85	92.4
HNT-ICG_10	8.79	87.9

**Figure 3:**
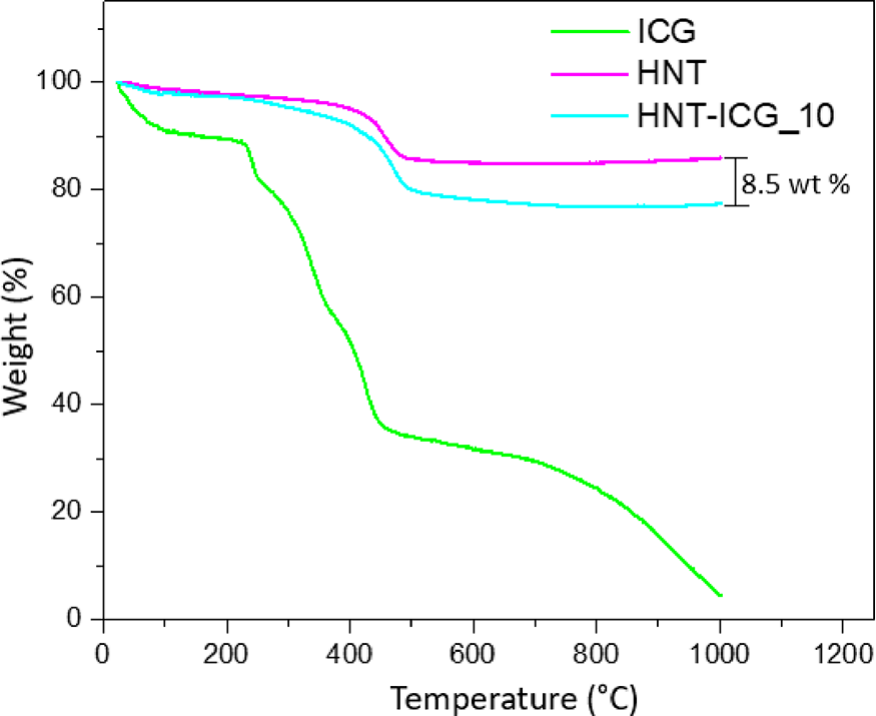
TGA of ICG, HNTs, and HNT-ICG_10 nanohybrids.

The ICG loading ratio of HNT-ICG_10 nanohybrids was further examined with TGA (Figure 3). Since ICG was completely decomposed after 1000 °C, the ICG loading ratio was calculated by the difference between the total weight loss between HNTs and the HNT-ICG_10 nanohybrids at 1000 °C. The TGA illustrated that the HNTs were loaded with ICG by 8.5 wt %, which coincided with the loading ratio obtained by the absorbance analysis. While there is no evidence of which is the dominant form of association, HNTs have been loaded into the lumen or adsorbed on the outer surface, resulting in successfully prepared HNT-ICG nanohybrids.

**Figure 4:**
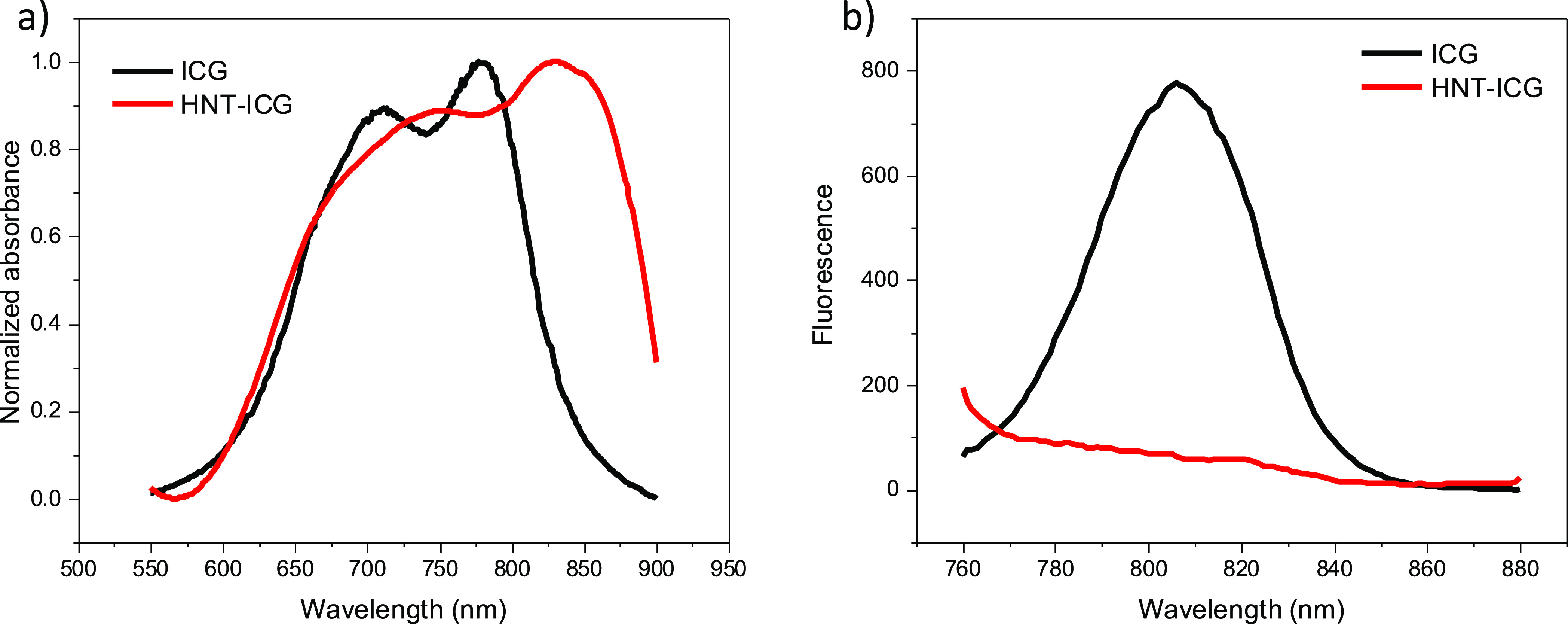
a) Absorbance spectra of 0.025 mg/mL aqueous ICG solution and 0.25 mg/mL aqueous HNT-ICG dispersion. b) Fluorescence spectra of 0.025 mg/mL aqueous ICG solution and 0.25 mg/mL aqueous HNT-ICG dispersion.

The HNT-ICG nanohybrids were further investigated in terms of their spectral properties to elucidate the noncovalent interactions between the ICG molecules and the HNT template and thus the mechanistic details of the loading process. Figure 4a presents the normalized absorbance spectra of the free ICG molecules in water and aqueous HNT-PDA dispersions. There has been a 60 nm bathochromic shift in the absorbance spectrum of the free ICG molecules when they were loaded into HNTs, which is indicative of J-like aggregates.^37,38^ The ICG molecules might have formed J-like assemblies via hydrophobic interactions that are stabilized with the HNTs. Free ICG molecules also showed dramatic fluorescence quenching when they formed nanohybrids with HNTs, potentially due to intramolecular charge transfer and intermolecular π–π stacking, further confirming the strong noncovalent interactions in the HNT-ICG nanohybrids (Figure 4b). This result also demonstrated that the singlet radiative decay pathway was inhibited and the nonradiative photothermal transition is the only decay pathway available for the HNT-ICG nanohybrids, making HNT-ICG nanohybrids effective photothermal agents.

**Figure 5:**
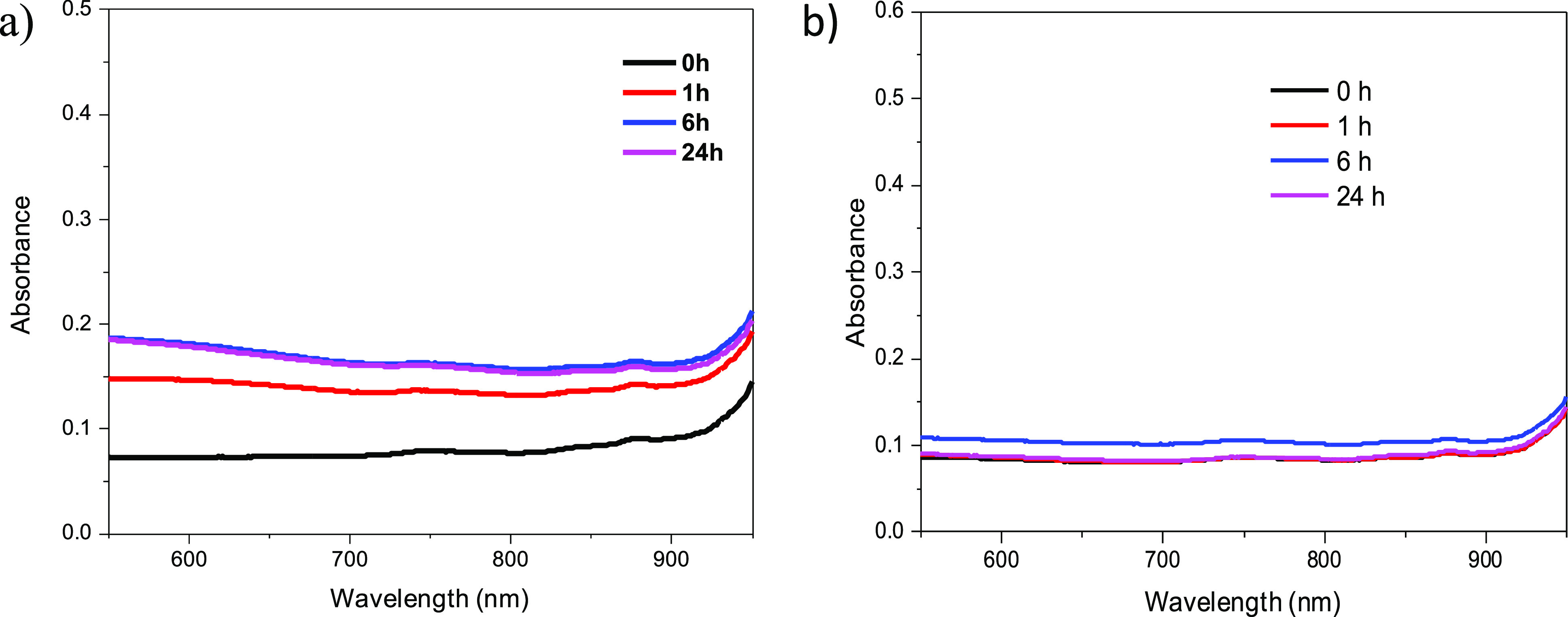
Release of ICG from HNT-ICG_10 nanohybrids in water (a) and DMF solvent (b).

Whether the ICG molecules are being released from the HNTs in an aqueous solution was studied by measuring the absorbance of the solution from which the HNT-ICG nanohybrids were removed by centrifugation at certain time intervals (Figure 5). The amount of ICG released from the nanohybrids was demonstrated to be negligible, demonstrating that ICG was not being released from the HNT-ICG nanohybrids when incubated in an aqueous solution for 24 h. Similarly, no significant release of ICG from the HNT-ICG nanohybrids was observed when the nanohybrids were incubated in DMF. Apparently, the strong hydrophobic interactions within the J-like assemblies of the ICG molecules and between ICG molecules and the HNT template were stabilized in the polar solvent and prevented the release of ICG.

**Figure 6:**
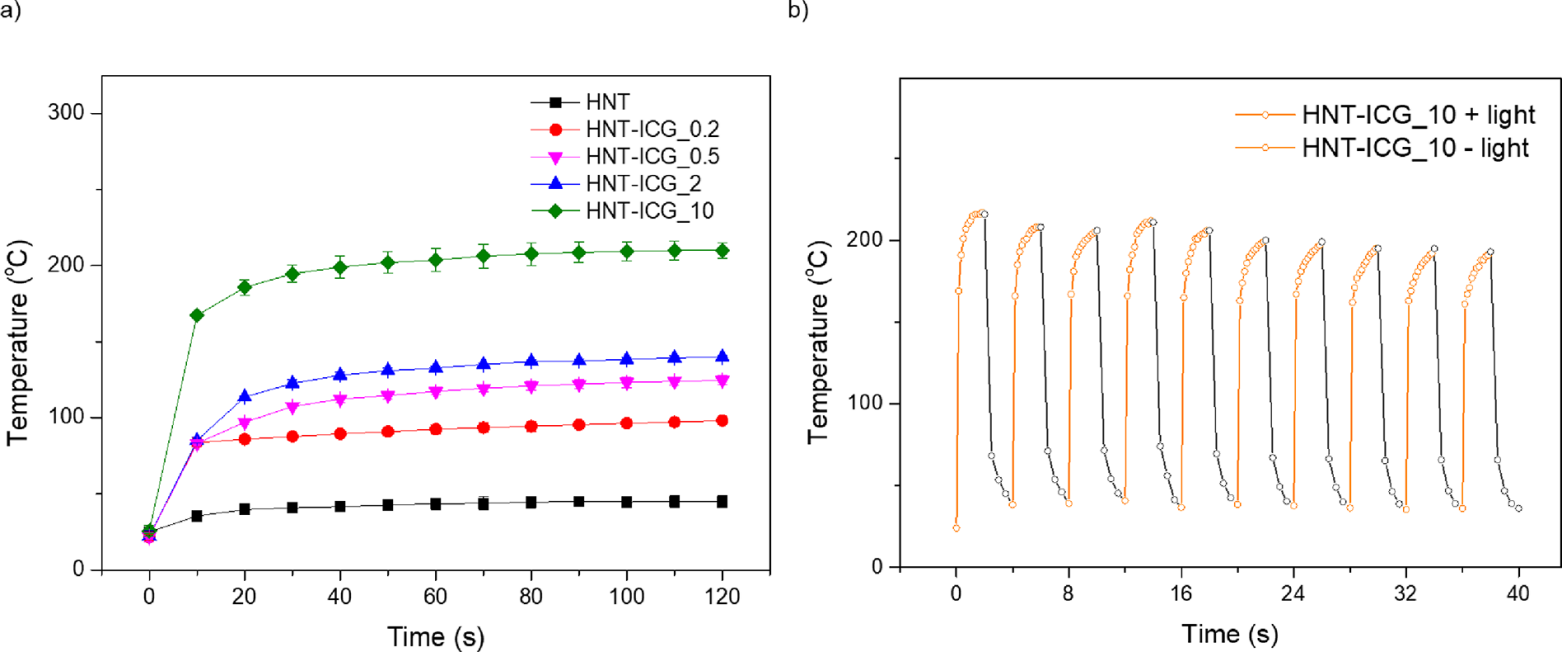
Time–temperature profiles of (a) HNT-ICG nanohybrids under irradiation from a laser module at 0.8 W/cm^2^ light density and (b) 10-cycle irradiation of HNT-ICG_10 nanohybrids.

The light-activated heating properties of HNT-ICG nanohybrids in powder form were investigated by constructing time–temperature profiles under NIR irradiation from an 808 nm laser module at a 0.8 W/cm^2^ light density (Figure 6a). While irradiation of HNTs under NIR light source did not lead to a significant temperature elevation, HNT-ICG nanohybrids heated up significantly under the same conditions due to the photothermal character of the loaded ICG molecules. The light-activated temperature elevations were observed to be in direct proportion to the photothermal agent loading rates of the nanohybrids. The temperature of the HNT-ICG_10 sample, which has the highest ICG content (10 wt %), was recorded to be 216 °C after 2 min NIR laser light irradiation. HNT-ICG_0.2 and HNT-ICG_0.5 nanohybrids demonstrated temperature increases above 100 °C despite containing only 0.2 and 0.5 wt % ICG, respectively. Hence, it has been proven that the HNT-ICG nanohybrids presented strong photothermal activity in powder form, which can be controlled by the amount of loaded ICG. Furthermore, the photothermal properties of the HNT-ICG nanohybrids were shown to be stable over 10 irradiation on/off cycles, as the light-activated temperature elevations obtained by HNT-ICG_10 nanohybrids decreased by only 7.4% at the end of 10 cycles (Figure 6b).

**Figure 7:**
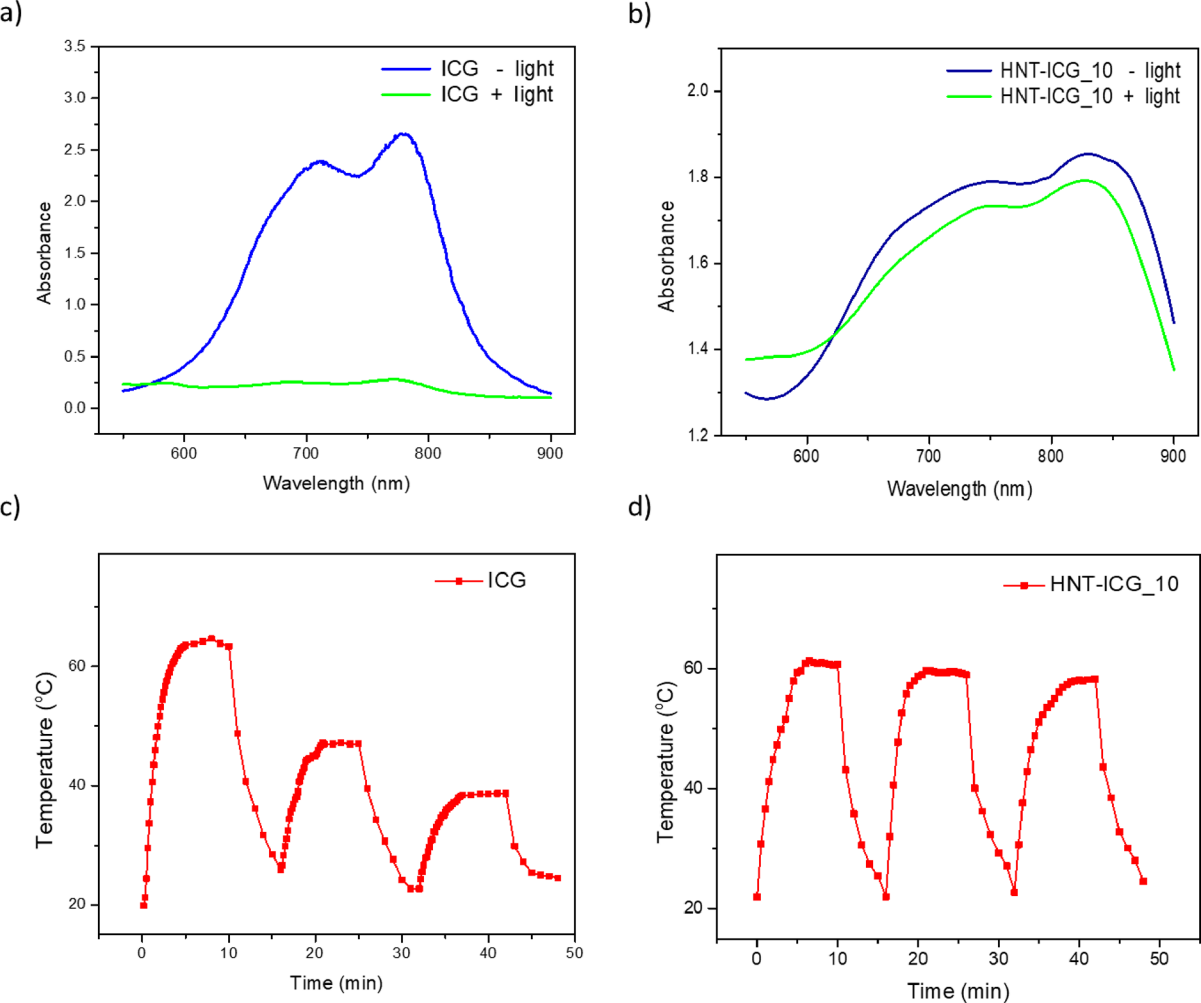
(a) Absorbance of 0.025 mg/mL aqueous ICG solution before and after three cycles of 10 min NIR irradiation. (b) Absorbance of aqueous 0.25 mg/mL HNT-ICG_10 dispersion before and after three cycles of 10 min NIR irradiation. Time temperature profiles of the aqueous ICG solution (c) and HNT-ICG nanohybrids (d) during three cycles of 10 min laser light irradiation followed by 6 min dark incubation.

Due to the limitations of ICG such as photodegradation, and instability in aqueous solutions when exposed to light, potential application areas are limited.^24^ In this view, the stability of the ICG has been the subject of several investigations in the literature.^39,40^ Here, the stabilization effect of HNTs on free ICG molecules was investigated. The strong absorption of free ICG molecules at 780 nm disappeared when the ICG solution was exposed to three cycles of 10 min of laser light irradiation followed by dark incubation for 6 min (Figure 7a). Thus, free ICG molecules were degraded after three cycles of NIR irradiation. The photobleaching of ICG seems to have occurred via a type II process involving ^1^O_2_-mediated dioxetane formation and dioxetane cleavage resulting in carbonyl products, where a type I process involving oxygen radicals and oxygen radical ions may also have contributed.^41,42^ On the other hand, when the ICG molecules formed nanohybrids with HNTs, after three NIR irradiation cycles, there was no noticeable difference between the absorbance spectra of HNT-ICG nanohybrids before and after NIR irradiation (Figure 7b). The fact that ICG molecules were protected from degradation under light when they were loaded in HNTs was further demonstrated by the photothermal stability of the HNT-ICG nanohybrids. The light-activated heating of the aqueous ICG solution decreased by 47% after the third irradiation cycle (Figure 7c) as a result of the degradation of the ICG molecules. On the other hand, HNT-ICG_10 nanohybrids that were heated to 60.7 °C at the end of the first irradiation cycle heated to 58.3 °C in the third cycle, presenting only a 3.95% decrease in temperature elevations after three cycles. The results revealed that, when loaded into HNTs, ICG molecules presented photothermal stability, and the resulting HNT-ICG nanohybrids can be reused in applications requiring multiple light-activated heating cycles (Figure 7d). HNTs served as a carrier that isolated the light-harvesting ICG molecules from environmental oxygen and protected them from oxidation and further decomposition, thereby maintaining their photophysical and photochemical properties.

**Figure 8:**
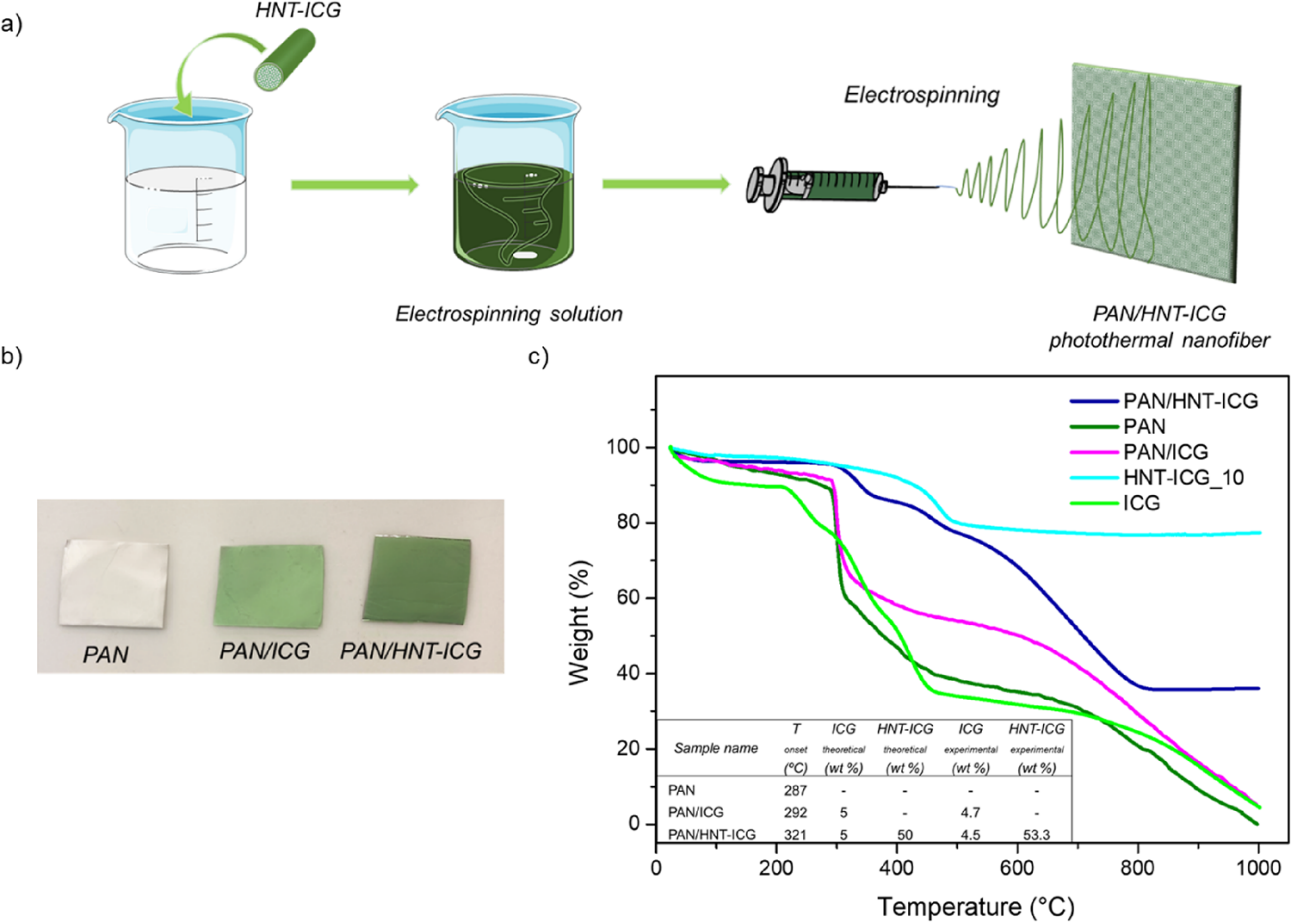
(a) Schematic representation of electrospinning of the PAN/HNT-ICG nanofibers. (b) TGA of HNT-ICG nanohybrids and PAN, PAN/ICG, and PAN/HNT-ICG nanofibers. (c) Photographs of PAN, PAN/ICG, and PAN/HNT-ICG nanofibers.

As a demonstration of the potential applications of highly stable HNT-ICG nanohybrids in photothermal applications, they have been incorporated into PAN nanofibers. Nanoparticles can be incorporated into polymeric nanofibers using the electrospinning method that creates nanofibers by using high voltage to create an electrically charged field and allowing the polymer to scatter in the form of fibers and accumulate in the collector. With their unique properties like being low cost and easy to manufacture, electrospun nanofibers are being widely utilized as filtration membranes, wound dressings, breathable membrane coatings, and coatings.^43^ Here, the incorporation of the HNT-ICG nanohybrids into nanofibers would allow the production of ICG-containing nanofibers in which the ICG molecules preserve their photothermal stability. The electrospinning technique was used to integrate HNT-ICG photothermal nanohybrids into the nanofiber in order to create composite nanofibers (Figure 8a). As a control, ICG was directly incorporated into the PAN electrospinning solution, resulting in PAN/ICG nanofibers, which did not contain the HNT nanocarriers. The produced nanofibers are notable for having a green tinge due to the characteristic color of ICG (Figure 8b). The PAN nanofiber’s white color was converted to green in the PAN/ICG and PAN/HNT-ICG nanofibers when ICG and HNT-ICG nanohybrids were incorporated. Figure 8c presents the TGA of ICG and PAN, PAN/ICG, and PAN/HNT-ICG nanofibers between 30 and 1000 °C. PAN/HNT-ICG nanofibers presented higher decomposition onset temperatures than neat PAN nanofibers, demonstrating the successful integration of the HNT-ICG nanohybrids and their strong interactions with the PAN polymer matrix. The fact that the theoretical and experimental amounts of ICG in the nanofibers were in good agreement further confirmed that PAN/HNT-ICG composite nanofibers were successfully synthesized.

**Figure 9:**
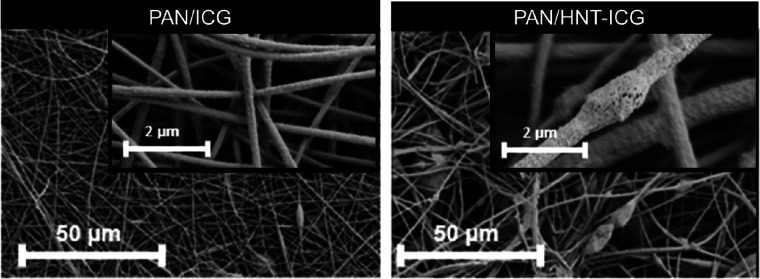
SEM images of PAN/ICG (left) and PAN/HNT-ICG (right) nanofibers.

The morphologies of PAN/HNT-ICG and PAN/ICG nanofibers were analyzed with SEM. Images in Figure 9 demonstrate that nanofiber formation and photothermal agent integration were accomplished successfully. In PAN/HNT-ICG composite nanofibers, in addition to nanofibers of different diameters, agglomerated particles and beads were observed. The agglomerated particles and beads are potentially composed of HNT-ICG photothermal nanohybrids that have not been completely dispersed in the electrospinning solution. Unlike the PAN/HNT-ICG nanofibers, no bead and agglomerated particles were observed in PAN/ICG nanofibers, because ICG molecules were thoroughly dissolved in the electrospinning solution.

**Figure 10:**
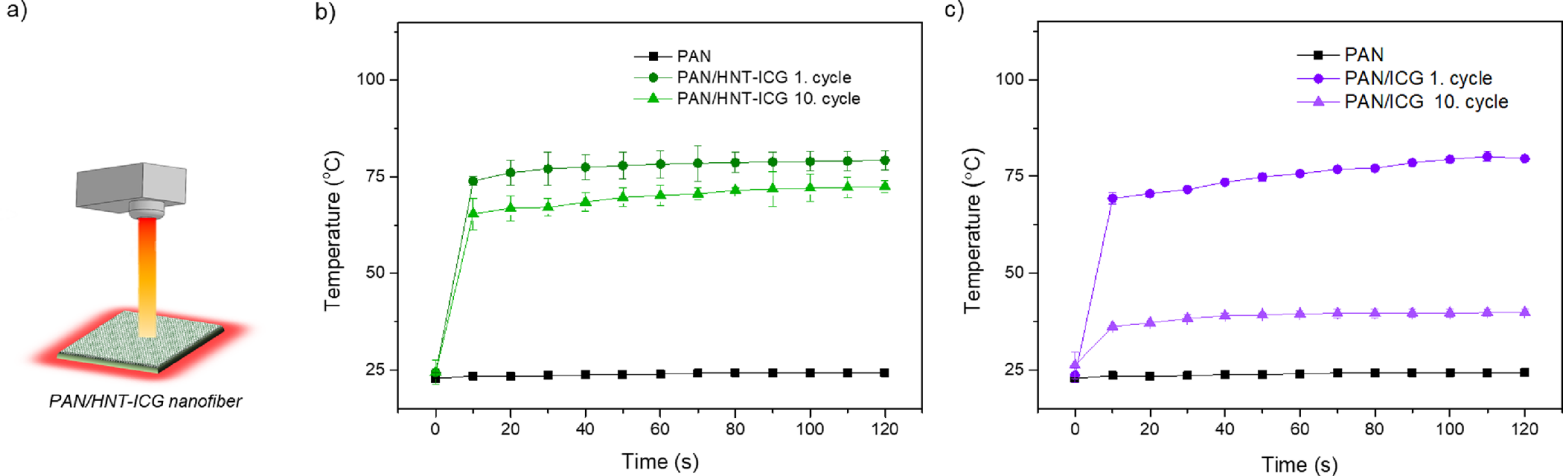
(a) Schematic representation of photothermal nanofiber irradiation. Time–temperature profiles of PAN/HNT-ICG nanofibers (b) and PAN/ICG nanofibers (c) before and after 10 cycles of 2 min irradiation from a NIR laser at 0.8 W/cm^2^ light density.

The photothermal properties of the PAN/HNT-ICG nanofibers were studied by monitoring their temperature increases under NIR irradiation (Figure 10a). While the neat PAN nanofiber did not heat up when irradiated with a NIR laser for 2 min, PAN/HNT-ICG nanofibers heated to 79.3 °C under the same conditions, demonstrating that the light-to-heat conversion properties of the HNT-ICG nanohybrids were preserved within the nanofiber (Figure 10b). When the irradiation of the same nanofiber was repeated for 10 cycles, the nanofibers were still able to heat up to 67.7 °C under NIR irradiation. The control PAN/ICG nanofibers, which were prepared by the incorporation of the ICG molecules directly into the PAN nanofiber without the HNT nanocarriers, were heated up to the same temperature as the PAN/HNT-ICG nanofibers in one NIR irradiation cycle. However, after 10 cycles of NIR laser light irradiation, the temperature they can reach was significantly lower, demonstrating that the free ICG molecules directly incorporated into nanofibers were decomposed upon light exposure (Figure 10c). This result confirmed that HNTs acted as nanocarriers that stabilize ICG molecules.

**Figure 11:**
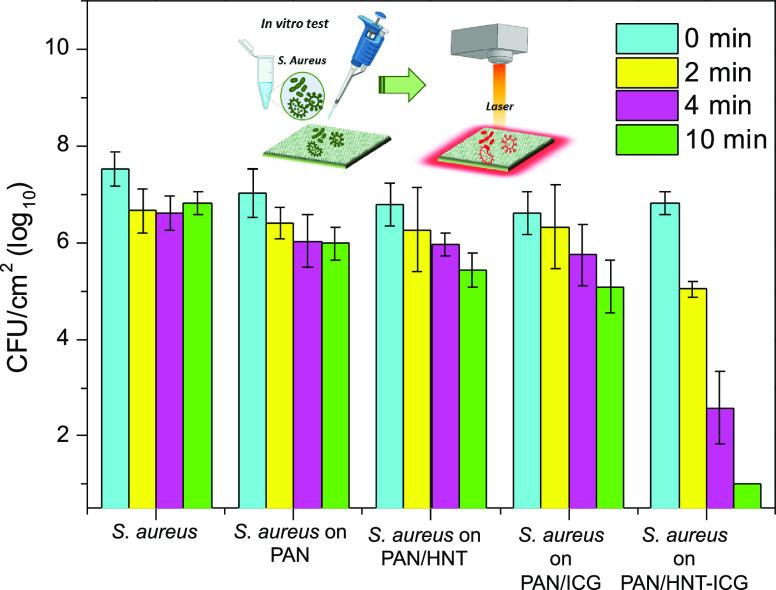
Viability of **S. aureus** suspensions alone and dropped on PAN, PAN/HNT, PAN/ICG, and PAN/HNT-ICG nanofibers under 0, 2, 4, and 10 min irradiation with an incandescent NIR light at 0.8 W/cm^2^ irradiance.

The ability of PAN/HNT-ICG photothermal composite nanofibers to kill bacteria upon NIR irradiation was investigated. The nanofibers were tested to see whether they can heat to temperatures that would cause disruption of bacterial cells via hyperthermia effects by NIR irradiation. After NIR irradiation of the nanofibers for various durations, the viability of the aqueous **S. aureus** suspension dropped on the composite nanofibers was investigated (Figure 11). Regardless of the irradiation time, irradiation of neat PAN and PAN/HNT nanofibers and nanofibers containing free ICG molecules resulted in negligible bacterial death. The number of viable **S. aureus** observed in the PAN/HNT-ICG nanofiber, on the other hand, decreased significantly with increasing NIR irradiation times. The PAN/HNT-ICG nanofiber killed all of the bacteria on its surface when exposed to irradiation for 10 min. The fact that the PAN polymer matrix does not absorb NIR light allowed the laser light to penetrate deeply into the nanofiber and remotely heat the photothermal agents embedded within the nanofiber. **S. aureus** suspensions exposed to the same irradiation conditions did not show any reduction in the number of viable bacteria, confirming that the bacteria were killed by light-activated heating of the HNT-ICG photothermal agents in the nanofiber rather than by direct light. Similarly, PAN/HNT nanofibers containing the same amount of HNTs as in the PAN/HNT-ICG nanofibers also did not present any significant killing on **S. aureus** bacteria either when irradiated or in the dark, indicating that bacteria–nanoparticle interactions also did not play a role in the mortality of the bacteria. The fact that the PAN nanofibers containing free ICG molecules were unable to kill the bacteria after 10 min demonstrated that the ICG molecules were degraded under light irradiation and lost their photothermal activity. On the other hand, when ICG molecules were encapsulated in HNTs, the resulting nanofibers preserved the photothermal activity of the ICG molecules. In conclusion, it has been demonstrated that PAN/HNT-ICG composite nanofibers have remarkable light-activated antibacterial properties and have a strong potential in applications requiring remote heating.

## Conclusions

ICG, an organic dye with unique photothermal conversion properties, was loaded into HNTs, clay-based natural nanoparticles. The free ICG, which is unstable in an aqueous environment, was stabilized against NIR irradiation when loaded into HNTs. In order to investigate the effect of the amount of ICG on the light-activated temperature elevations, different ICG–HNT ratios were tested. An increase in the temperature was seen in all of them as a result of NIR irradiation. After being exposed to NIR laser light for 2 min, the HNT–ICG nanohybrid’s local temperature rises and maintains its stability over multiple irradiation cycles. The nanohybrid with the highest photothermal activity, HNT-ICG_10 nanohybrid, was successfully converted into nanofiber form using the electrospinning method. The resulting composite nanofibers were demonstrated to be heated significantly upon NIR exposure and kill bacteria they are in contact with.

## References

[ref1] Tee S. Y.; Win K. Y.; Goh S. S.; Teng C. P.; Tang K. Y.; Regulacio M. D.; Li Z.; Ye E. Chapter 1: Introduction to Photothermal Nanomaterials. In RSC Nanoscience and technology; 2022; Vol. 2022-January, pp 1–32. 10.1039/9781839165177-00001.

[ref2] Austin L. A.; MacKey M. A.; Dreaden E. C.; El-Sayed M. A. The Optical, Photothermal, and Facile Surface Chemical Properties of Gold and Silver Nanoparticles in Biodiagnostics, Therapy, and Drug Delivery. Arch. Toxicol. 2014, 88, 1391–1417. 10.1007/s00204-014-1245-3. 24894431 PMC4136654

[ref3] Cobley C. M.; Au L.; Chen J.; Xia Y. Targeting Gold Nanocages to Cancer Cells for Photothermal Destruction and Drug Delivery. Expert Opin. Drug Delivery 2010, 29, 577–587. 10.1517/17425240903571614. PMC2858262 20345327

[ref4] Tao B.; Lin C.; Deng Y.; Yuan Z.; Shen X.; Chen M.; He Y.; Peng Z.; Hu Y.; Cai K. Copper-Nanoparticle-Embedded Hydrogel for Killing Bacteria and Promoting Wound Healing with Photothermal Therapy. J. Mater. Chem. B 2019, 7 (15), 2534–2548. 10.1039/C8TB03272F. 32255130

[ref5] Wang P. Emerging Investigator Series: The Rise of Nano-Enabled Photothermal Materials for Water Evaporation and Clean Water Production by Sunlight. Environ. Sci. Nano 2018, 5 (5), 1078–1089. 10.1039/C8EN00156A.

[ref6] Liu Y.; Li F.; Guo Z.; Xiao Y.; Zhang Y.; Sun X.; Zhe T.; Cao Y.; Wang L.; Lu Q.; Wang J. Silver Nanoparticle-Embedded Hydrogel as a Photothermal Platform for Combating Bacterial Infections. Chem. Eng. J. 2020, 382, 122990 10.1016/j.cej.2019.122990.

[ref7] McGrath A. J.; Chien Y. H.; Cheong S.; Herman D. A. J.; Watt J.; Henning A. M.; Gloag L.; Yeh C. S.; Tilley R. D. Gold over Branched Palladium Nanostructures for Photothermal Cancer Therapy. ACS Nano 2015, 9 (12), 12283–12291. 10.1021/acsnano.5b05563. 26549201

[ref8] Hessel C. M.; Pattani V. P.; Rasch M.; Panthani M. G.; Koo B.; Tunnell J. W.; Korgel B. A. Copper Selenide Nanocrystals for Photothermal Therapy. Nano Lett. 2011, 11 (6), 2560–2566. 10.1021/nl201400z. 21553924 PMC3111000

[ref9] Manikandan M.; Hasan N.; Wu H. F. Platinum Nanoparticles for the Photothermal Treatment of Neuro 2A Cancer Cells. Biomaterials 2013, 34 (23), 5833–5842. 10.1016/j.biomaterials.2013.03.077. 23642996

[ref10] Oruc B.; Unal H. Fluorophore-Decorated Carbon Nanotubes with Enhanced Photothermal Activity as Antimicrobial Nanomaterials. ACS Omega 2019, 4 (3), 5556–5564. 10.1021/acsomega.9b00099.

[ref11] Dao V.-D.; Choi H.-S. Carbon-Based Sunlight Absorbers in Solar-Driven Steam Generation Devices. Global Challenges 2018, 2 (2), 1700094 10.1002/gch2.201700094. 31565322 PMC6607331

[ref12] Dash B. S.; Jose G.; Lu Y. J.; Chen J. P. Functionalized Reduced Graphene Oxide as a Versatile Tool for Cancer Therapy. Int. J. Mol. Sci. 2021, 22 (6), 2989 10.3390/ijms22062989. 33804239 PMC8000837

[ref13] Yuce S.; Demirel O.; Alkan Tas B.; Sungur P.; Unal H. halloysite Nanotube/Polydopamine nanohybrids as Clay-Based Photothermal Agents for Antibacterial Applications. ACS Appl. Nano Mater. 2021, 4 (12), 13432–13439. 10.1021/acsanm.1c02936.

[ref14] Yang J.; Choi J.; Bang D.; Kim E.; Lim E. K.; Park H.; Suh J. S.; Lee K.; Yoo K. H.; Kim E. K.; Huh Y. M.; Haam S. Convertible Organic Nanoparticles for Near-Infrared Photothermal Ablation of Cancer Cells. Angew. Chem. Int. Ed. 2011, 50 (2), 441–444. 10.1002/anie.201005075. 21132823

[ref15] Sheng Z.; Hu D.; Xue M.; He M.; Gong P.; Cai L. Indocyanine Green Nanoparticles for Theranostic Applications. Nano-Micro Lett. 2013, 5 (3), 145–150. 10.5101/nml.v5i3.p145-150.

[ref16] Chen Y.; Li L.; Chen W.; Chen H.; Yin J. Near-Infrared Small Molecular Fluorescent Dyes for Photothermal Therapy. Chin. Chem. Lett. 2019, 30 (7), 1353–1360. 10.1016/j.cclet.2019.02.003.

[ref17] Jonak C.; Skvara H.; Kunstfeld R.; Trautinger F.; Schmid J. A. Intradermal Indocyanine Green for in Vivo Fluorescence Laser Scanning Microscopy of Human Skin: A Pilot Study. PLoS One 2011, 6 (8), e23972 10.1371/journal.pone.0023972. 21904601 PMC3164142

[ref18] Song X.; Chen Q.; Liu Z. Recent Advances in the Development of Organic Photothermal Nano-Agents. Nano Res. 2015, 8, 340–354. 10.1007/s12274-014-0620-y.

[ref19] Reinhart M. B.; Huntington C. R.; Blair L. J.; Heniford B. T.; Augenstein V. A. Indocyanine Green:Historical Context, Current Applications, and Future Considerations. Surg. Innovation 2016, 23 (2), 166–175. 10.1177/1553350615604053. 26359355

[ref20] Shramova E. I.; Kotlyar A. B.; Lebedenko E. N.; Deyev S. M.; Proshkina G. M. Near-Infrared Activated Cyanine Dyes As Agents for Photothermal Therapy and Diagnosis of Tumors. Acta Nat. 2020, 27, 102–113. 10.32607/actanaturae.11028. PMC7604893 33173600

[ref21] Bertani C.; Cassinotti E.; Della Porta M.; Pagani M.; Boni L.; Baldari L. Indocyanine Green-a Potential to Explore: Narrative Review. Ann. Laparosc. Endosc. Surg. 2022, 7, 1–12. 10.21037/ales-21-5.

[ref22] Alander J. T.; Kaartinen I.; Laakso A.; Pätilä T.; Spillmann T.; Tuchin V. V.; Venermo M.; Välisuo P. A Review of Indocyanine Green Fluorescent Imaging in Surgery. Int. J. Biomed. Imaging 2012, 2012, 7 10.1155/2012/940585. PMC3346977 22577366

[ref23] He S.; Song J.; Qu J.; Cheng Z. Crucial Breakthrough of Second Near-Infrared Biological Window Fluorophores: Design and Synthesis toward Multimodal Imaging and Theranostics. Chem. Soc. Rev. 2018, 47 (12), 4258–4278. 10.1039/C8CS00234G. 29725670

[ref24] Saxena V.; Sadoqi M.; Shao J. Enhanced Photo-Stability, Thermal-Stability and Aqueous-Stability of Indocyanine Green in Polymeric Nanoparticulate Systems. J. Photochem. Photobiol. B 2004, 74, 29–38. 10.1016/j.jphotobiol.2004.01.002. 15043844

[ref25] Saxena V.; Sadoqi M.; Shao J. Degradation Kinetics of Indocyanine Green in Aqueous Solution. J. Pharm. Sci. 2003, 92 (10), 2090–2097. 10.1002/jps.10470. 14502548

[ref26] Yu J.; Yaseen M. A.; Anvari B.; Wong M. S. Synthesis of Near-Infrared-Absorbing Nanoparticle-Assembled Capsules. Chem. Mater. 2007, 19 (6), 1277–1284. 10.1021/cm062080x.

[ref27] Kirchherr A. K.; Briel A.; Mäder K. Stabilization of Indocyanine Green by Encapsulation within Micellar Systems. Mol. Pharm. 2009, 6 (2), 480–491. 10.1021/mp8001649. 19228053

[ref28] Gowsalya K.; Yasothamani V.; Vivek R. Emerging Indocyanine Green-Integrated Nanocarriers for Multimodal Cancer Therapy: A Review. Nanoscale Adv. 2021, 3, 3332–3352. 10.1039/D1NA00059D. 36133722 PMC9418715

[ref29] Zheng X.; Zhou F.; Wu B.; Chen W. R.; Xing D. Enhanced Tumor Treatment Using Biofunctional Indocyanine Green-Containing Nanostructure by Intratumoral or Intravenous Injection. Mol. Pharm. 2012, 9 (3), 514–522. 10.1021/mp200526m. 22332810 PMC3418867

[ref30] Liu C.; Ruan C.; Shi R.; Jiang B. P.; Ji S.; Shen X. C. A near Infrared-Modulated thermosensitive Hydrogel for Stabilization of Indocyanine Green and Combinatorial Anticancer Phototherapy. Biomater. Sci. 2019, 7 (4), 1705–1715. 10.1039/C8BM01541D. 30758351

[ref31] Qiu H.; Zhu S.; Pang L.; Ma J.; Liu Y.; Du L.; Wu Y.; Jin Y. ICG-Loaded Photodynamic Chitosan/Polyvinyl Alcohol Composite Nanofibers: Anti-Resistant Bacterial Effect and Improved Healing of Infected Wounds. Int. J. Pharm. 2020, 588, 119797 10.1016/j.ijpharm.2020.119797. 32828977

[ref32] Massaro M.; Lazzara G.; Noto R.; Riela S. halloysite Nanotubes: A Green Resource for Materials and Life Sciences. Rendiconti Lincei. Scienze Fisiche e Naturali 2020, 31 (2), 213–221. 10.1007/s12210-020-00886-x.

[ref33] Yuan P.; Tan D.; Annabi-bergaya F. Applied Clay Science Properties and Applications of halloysite Nanotubes: Recent Research Advances and Future Prospects. Appl. Clay Sci. 2015, 112–113, 75–93. 10.1016/j.clay.2015.05.001.

[ref34] Kausar A. Review on Polymer/halloysite Nanotube Nanocomposite. Polym. - Plast. Technol. Eng. 2018, 57 (6), 548–564. 10.1080/03602559.2017.1329436.

[ref35] Hendessi S.; Sevinis E. B.; Unal S.; Cebeci F. C.; Menceloglu Y. Z.; Unal H. Antibacterial Sustained-Release Coatings from halloysite Nanotubes/Waterborne Polyurethanes. Prog. Org. Coat. 2016, 101, 253–261. 10.1016/j.porgcoat.2016.09.005.

[ref36] Landsman M. L. J.; Kwant G.; Mook G. A.; Zijlstra W. G. Light Absorbing Properties, Stability, and Spectral Stabilization of Indocyanine Green. J. Appl. Physiol. 1976, 40 (4), 575–583. 10.1152/jappl.1976.40.4.575. 776922

[ref37] Liu R.; Tang J.; Xu Y.; Zhou Y.; Dai Z. Nano-Sized Indocyanine Green j-Aggregate as a One-Component Theranostic Agent. Nanotheranostics 2017, 1 (4), 430–439. 10.7150/ntno.19935. 29188176 PMC5704008

[ref38] Cheung C. C. L.; Ma G.; Karatasos K.; Seitsonen J.; Ruokolainen J.; Koffi C. R.; Hassan H. A. F. M.; Al-Jamal W. T. Liposome-Templated Indocyanine Green J-Aggregates for in Vivo near-Infrared Imaging and Stable Photothermal Heating. Nanotheranostics 2020, 4 (2), 91–106. 10.7150/ntno.41737. 32190536 PMC7064739

[ref39] Li L.; Liu Y.; Hao P.; Wang Z.; Fu L.; Ma Z.; Zhou J. PEDOT Nanocomposites Mediated Dual-Modal Photodynamic and Photothermal Targeted Sterilization in Both NIR I and II Window. Biomaterials 2015, 41, 132–140. 10.1016/j.biomaterials.2014.10.075. 25522972

[ref40] Wang Y.; Xie Y.; Li J.; Peng Z. H.; Sheinin Y.; Zhou J.; Oupický D. Tumor-Penetrating Nanoparticles for Enhanced Anticancer Activity of Combined Photodynamic and Hypoxia-Activated Therapy. ACS Nano 2017, 11 (2), 2227–2238. 10.1021/acsnano.6b08731. 28165223 PMC5332348

[ref41] Gorka A. P.; Nani R. R.; Schnermann M. J. Cyanine Polyene Reactivity: Scope and Biomedical Applications. Org. Biomol. Chem. 2015, 13 (28), 7584–7598. 10.1039/C5OB00788G. 26052876 PMC7780248

[ref42] Holzer W.; Mauerer M.; Penzkofer A.; Szeimies R. M.; Abels C.; Landthaler M.; Bäumler W. Photostability and Thermal Stability of Indocyanine Green. J. Photochem. Photobiol. B 1998, 47 (2–3), 155–164. 10.1016/S1011-1344(98)00216-4. 10093915

[ref43] Islam M. S.; Ang B. C.; Andriyana A.; Afifi A. M. A Review on Fabrication of Nanofibers via Electrospinning and Their Applications. SN Appl. Sci. 2019, 20, 1248 10.1007/s42452-019-1288-4.

